# A Finite Element Analysis to Compare Stress Distribution on Extra-Short Implants with Two Different Internal Connections

**DOI:** 10.3390/jcm8081103

**Published:** 2019-07-25

**Authors:** Silvia Helena García-Braz, María Prados-Privado, Luiz Carlos Silveira Zanatta, José Luis Calvo-Guirado, Juan Carlos Prados-Frutos, Sérgio Alexandre Gehrke

**Affiliations:** 1 Department of Implantology, Paulista University, São Paulo 01000, Brazil; 2 Department of Continuum Mechanics and Structural Analysis, Carlos III University, Leganés, 28911 Madrid, Spain; 3 Asisa Dental SAU, 28912 Madrid, Spain; 4 Department of Oral and Implant Surgery, Universidad Católica de Murcia, 30107 Murcia, Spain; 5 Department of Medicine and Surgery, Rey Juan Carlos University, Alcorcón, 28933 Madrid, Spain; 6 Department of Research, Biotecnos—Technology and Science, Montevideo 11100, Uruguay

**Keywords:** von Mises stress, extra-short dental implant, hexagonal internal connection, morse taper connection

## Abstract

Background: The goal of this study was to analyze the stress distribution on two types of extra-short dental implants with 5 mm of length: An internal hexagon (IH) and morse taper connection (MT). Methods: The three-dimensional model was composed of trabecular and cortical bone, a crown, an extra-short dental implant and their components. An axial load of 150 N was applied and another inclined 30° with the same magnitude. Results: Stress concentrations on the IH implant are observed in the region of the first threads for the screw. However, in the MT implant the highest stress occurs at the edges of the upper implant platform. Conclusions: In view of the results obtained in this study the two types of prosthetic fittings present a good stress distribution. The Morse taper connections presented better behavior than the internal in both loading configurations.

## 1. Introduction

The posterior areas of the mandible are affected by reabsorption with a high degree of vertical bone loss, especially in a patient who maintains these areas without rehabilitating for a long time or using removable prostheses for a long period of time. Patients with bone atrophies or reduced bone height can have difficulties to perform treatments with conventional dental implants. In these circumstances, clinicians usually use more invasive and expensive techniques [1] such as bone grafting, bone regeneration, transposition of the dental nerve or the use of unconventional implants (tilted, transmandibular) [2,3]. An alternative to these invasive treatments are short implants, which have an important goal when a treatment in maxillary and mandible is necessary with a less invasive procedure and a good predictability [4].

Although there is no consensus about the implant length to be considered short or extra-short, this study will use the classification proposed by Al-Johany et al. where 6 mm or less of length is classified as extra-short [5]. The success of dental implants depends on variables like implant geometry, loading conditions and bone quality, among others [6]. Initially, the use of these implants was associated with low success rates [7]. However, changes in surface treatment and macrogeometry of implants, as well as the development of new surgical techniques and the better knowledge of biomechanics applied to short implants have increased success and survival. Some studies have reported the effect of roughness of the surface in failure rates [8] and they have concluded that these kinds of failures have a relation with the higher bone-to-implant contact [9]. Another factor in success rates is the crown-to-implant ratio, which plays an important role in dental implants treatment [10].

The use of extra-short dental implants can imply increasing the crown-to-implant ratios [11]. The European Academy of Osseointegration recommend and accept a crown-to-implant ratio of 2:1 [12]. Mechanically, the treatment with short or extra-short dental implants can present a high probability of failure due to the relation between the ratio of the crown and the size of the bone fixation [13]. Even several studies have concluded that the single crown supported by a short dental implant is a good option in the posterior region, and because of it there is a high rate of success and minimal bone loss [14,15].

Extra-short dental implants have nowadays survival rates similar to conventional dental implants when the number of dental implants and the antagonist are optimal [16,17,18,19,20]. The purpose of this study was to perform a finite element analysis to compare stress distribution in two extra-short dental implants with different connections (HI and MT) with a load of 150 N axial and an oblique load of 30°.

## 2. Materials and Methods

### 2.1. Extra-Short Dental Implants Analyzed

The geometric models of implants and components were supplied by the manufacturer (Implacil De Bortoli, São Paulo, Brazil).

**Figure 1:**
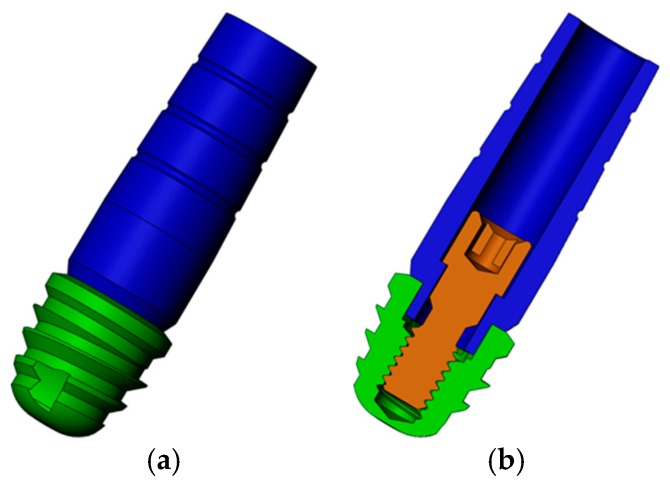
Three-dimensional model of internal hexagon (IH) extra-short implants: (**a**) Implant in green and abutment in blue; (**b**) transversal cut of the model to view the screw (brown).

Figure 1 details the implant with an internal hexagon connection (IH) with a diameter and a length of 5 mm.

**Figure 2:**
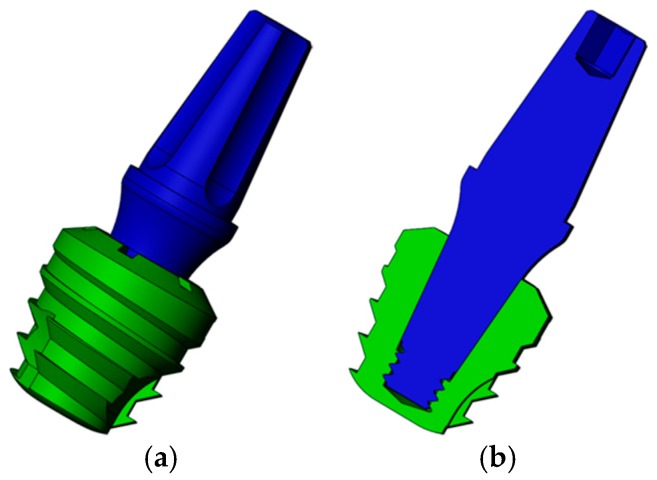
Three-dimensional model of Morse taper (MT) extra-short implants: (**a**) Implant in green and abutment in blue; (**b**) transversal cut of the model.

Figure 2 shows the implant with a Morse taper (MT) connection. This implant also has 5 mm of diameter and length.

### 2.2. Three-Dimensional Model

**Figure 3:**
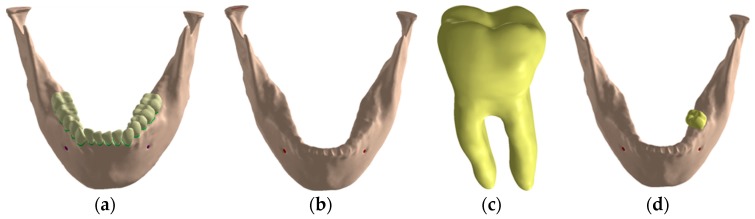
Jaw three-dimensional model: (**a**) Jaw with teeth; (**b**) edentulous jaw; (**c**) model of the extracted tooth; (**d**) tooth insertion in the edentulous mandible.

A three-dimensional mandible model was used in this study and obtained following the reconstruction steps described in a Vasco et al. study [21] (Figure 3a). The required geometric changes were performed in SolidWorks 2013 (Dassault Systemes, Solidworks Corps, Waltham, MA, USA). To decrease computational time, the models were edited to use edentulous jaw, along with the tooth model 36 of the jaw to provide the external geometry of the future implant prosthesis, as shown in Figure 3.

In addition to the jaw, all models will have the following characteristics:

- Osseointegrated variable implant, positioned in the posterior region of the mandible, at the site of element 3.6, using the external geometry of the reconstructed tooth in tomography for the construction of the prosthetic crown.
- Single metal-ceramic crown, supported and cemented implant, with infrastructure of cobalt chromium on the intermediate, with a minimum thickness of 0.3 mm and feldspathic porcelain covering over the infrastructure, with a minimum of 1 mm thickness.
- Zinc phosphate cement layer approximately 0.1 mm thick between crowns and intermediates [22,23,24,25].
- Intermediate variable, with a height of 7.5 mm between its upper portion and the implant platform.
- Structure to simulate the occlusal third of the enamel antagonist teeth for the axial load models. It presents circular points of contact with a mm of diameter, being three points of contact; in the vestibular cusp, with one in the buccal slope and one in the lingual slope and in the lingual cusp, with one in the buccal slope.
- Structure to standardize the application of the oblique load, positioned in the vestibular slopes of the lingual cusps.

### 2.3. Bone Loss Modelling

To simulate a condition of bone atrophy that justifies the use of the implants analyzed, a posterior unilateral wear was performed, keeping the implant closer to the inferior alveolar nerve at a distance of two mm. This distance was selected because it is the recommended minimum between the implant and inferior alveolar nerve to avoid nerve damage [26].

**Figure 4:**
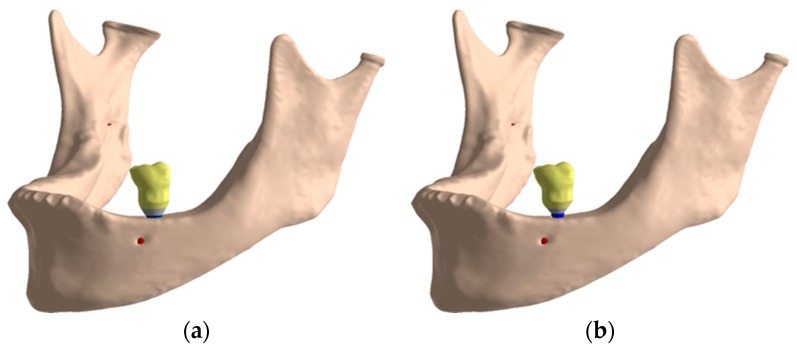
Final assembly: (**a**) IH connection; (**b**) MT connection.

**Figure 5:**
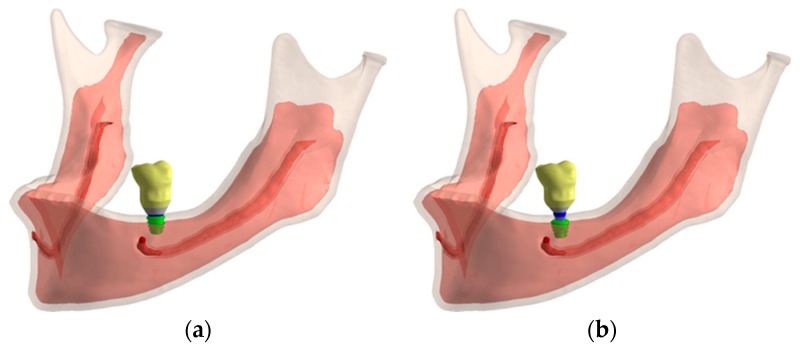
Cortical and trabecular bone geometry: (**a**) IH connection; (**b**) MT connection.

Once the bone model with the atrophy was obtained the assembly of the implant, the prosthetic components and the crown was placed in position 36, as Figure 4 shows. Figure 5 details the geometry of the cortical and trabecular bone with the implant and the crown.

### 2.4. Material Properties and Contact Conditions

All components were considered isotropic, homogeneous and linearly elastic. Table 1 details the Young’s modulus and the Poisson’s ratio assigned to each material.

Non-linear contacts of the frictional type were simulated to approximate the simulation to the real condition, with a coefficient of friction value of 0.2 to simulate the contact between the titanium structures [33]. The contacts between the infrastructure and zinc phosphate cement were also configured with a coefficient of friction of 0.2 [34,35], since there is no perfect adhesion between the zinc phosphate cement and other structures, but only mechanical enmeshment [36]. All other contacts were simulated as a perfect adhesion, except for contact in the axial antagonist, as explained in next section. Rigid supports were added in the areas of insertion of the jaw lift muscles. The simulations were non-linear in relation to the contact.

### 2.5. Loading Condition

The antagonist structure was modelled with frictionless supports on its sides to simulate occlusal contact, to allow only occlusal-gingival movement. The contacts between the antagonist structure and the crowns were configured without friction, which allows sliding and gaps formation.

**Figure 6:**
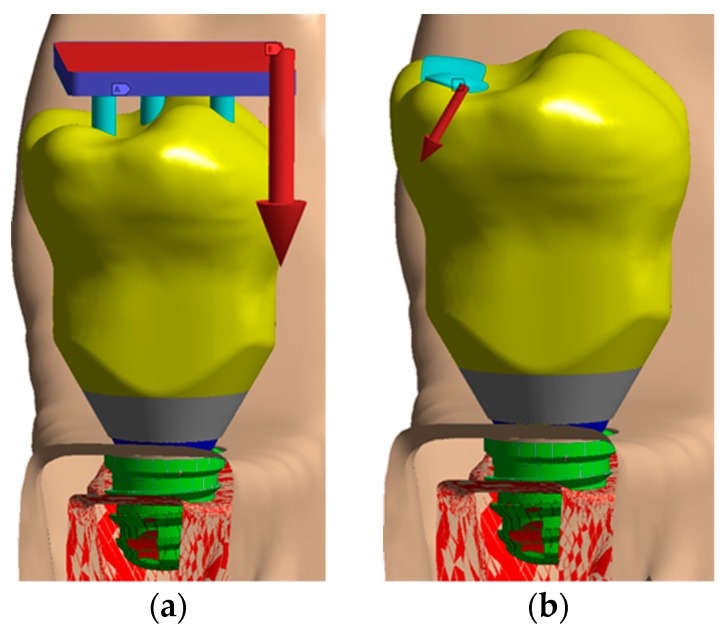
Loading configuration: (**a**) Axial load; (**b**) oblique load.

With the oblique loading, a vector load in the lingual-lingual direction was simulated with an angle of 30° as Figure 6b shows. Figure 6a details how the axial load was applied in the model.

### 2.6. Mesh Generation

**Figure 7:**
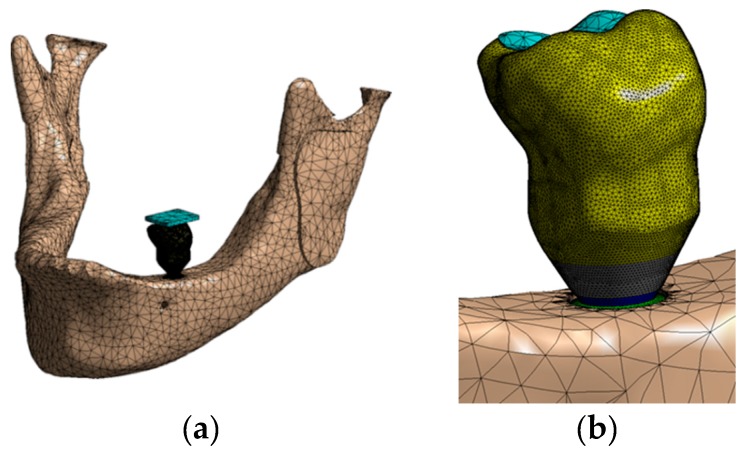

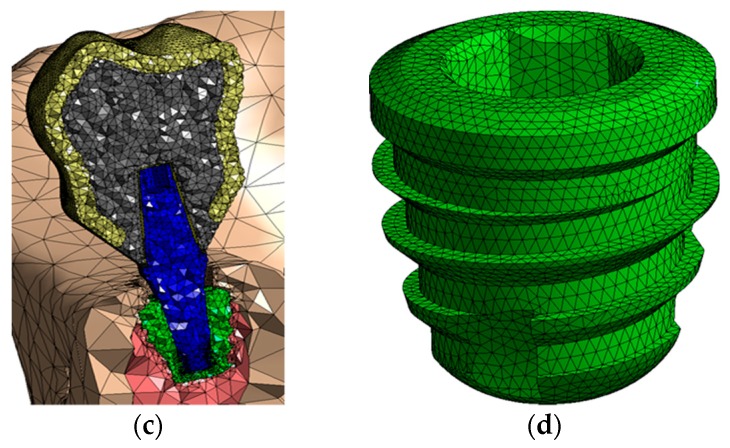
Mesh generated: (**a**) Whole assembly; (**b**) a view of the crown; (**c**) a transversal cut of the model; (**d**) IH implant.

The mesh was generated with tetrahedral quadratic elements (solid 187), allowing the copying of irregular geometry present in the analyzed models. The number of nodes/elements ranged from 648,367/389,916 to 753,110/439,923. Figure 7 shows some images of the meshes generated. All models were analyzed in a Windows 7 64-bit, Intel I7 920 processor, 24 Gb RAM. The convergence criterion was a change of less than 5% in von Mises stress in the model [37].

## 3. Results

### 3.1. Stress Distribution on Extra-Short Implants with an Axial Load

**Figure 8:**
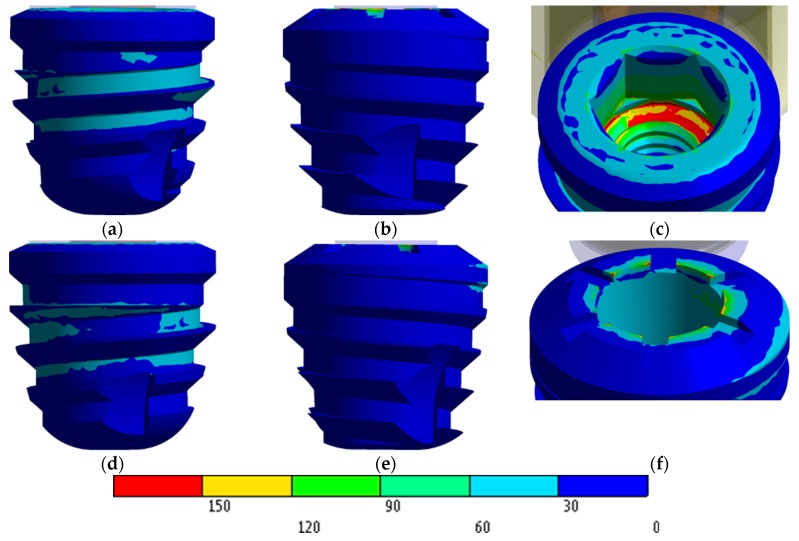
von Mises stress distribution in MPa: (**a**) IH vestibular view; (**b**) IH distal view; (**c**) IH stress concentration; (**d**) MT vestibular view; (**e**) MT distal view; (**f**) MT stress concentration.

Figure 8 shows the stress distribution on extra-short implants with an axial load. In view of that figure, the vestibular area has a bigger stress than the distal one.

Stress concentrations on the IH implant are observed in the region of the first threads for the screw (Figure 8c). However, in the MT implant the highest stress occurs at the edges of the upper implant platform (Figure 8f).

Regarding the IH extra-short implant, the internal hexagon is not employed to dissipate the stress caused by the load. In this case, stresses are dissipated through the internal threads of the implant, the implant-to-screw contact region, and the intermediate-implant contact region. However, the MT extra-short implant obtained a favorable and homogenous distribution along the contact area in the internal cone of the implant. The highest values appeared living angles formed between the platform and cone.

### 3.2. Stress Distribution on Extra-Short Implants with an Oblique Load

**Figure 9:**
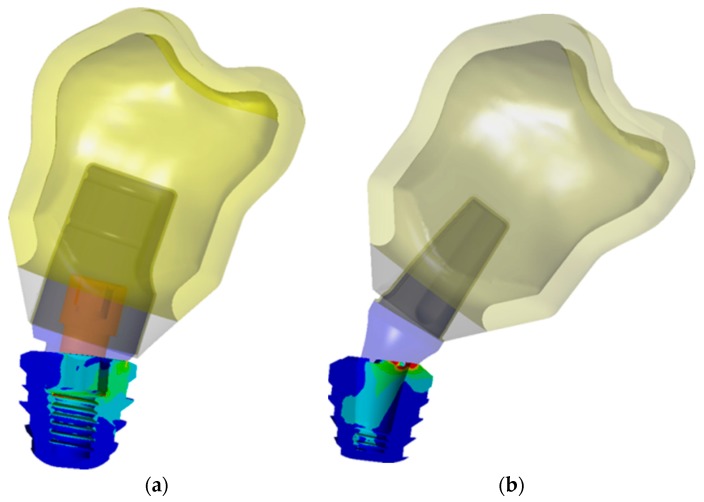
Stress distribution on implant with an oblique load: (**a**) IH connection; (**b**) MT connection.

Stress concentrations in the IH connection implant are observed both in the palatal portion of the implant platform and in the first internal threads in the vestibular region as shown in Figure 9a.

In the case of the MT implant, the concentrations occurred predominantly in the lingual region of the platform. Unlike the IH implant, the MT connection has a juxtaposition in the cone that evenly distributes the tensions in the middle and lower regions of the implant. However, this resulted in an accumulation of stresses in the angle between the platform and cone, not only by the presence of the live angle, but also because the locking of the MT connection caused a greater deformation of the intermediate above the contact region and therefore a stress concentration (Figure 9b).

### 3.3. Stress Distribution on Bone

**Figure 10:**
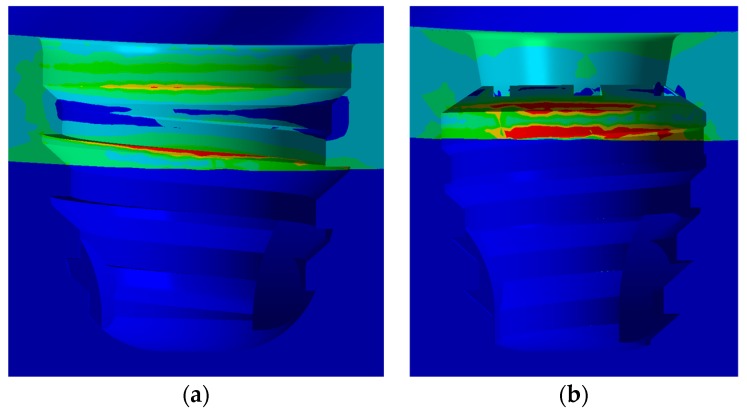

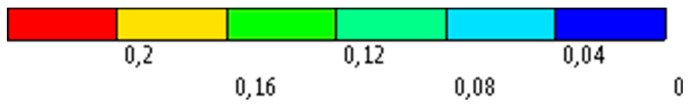
Stress distribution on bone with an axial load: (**a**) IH connection; (**b**) MT connection.

Figure 10 shows the stress distribution in the bone using the Mohr Coulomb scale. In view of that figure, both connections provide similar maximum stress values in the bone.

**Figure 11:**
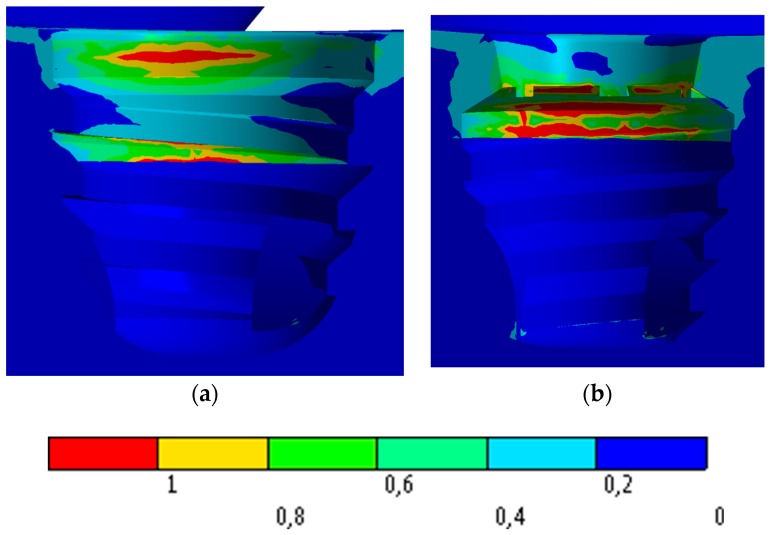
Stress distribution on bone with an oblique load: (**a**) IH connection; (**b**) MT connection.

Bone stress distribution with the oblique stress is detailed in Figure 11.

## 4. Discussion

The goal of this study was to analyze the stress distribution on two types of extra-short dental implants with 5 mm of length under the axial and oblique load of 150 N: An internal connection (IH) and Morse taper connection (MT). With the axial and oblique load, both connections obtained a maximum stress less than 520 MPa, which is the stress to obtain a plastic strain in the bone. This study has an assumption regarding the material properties because all of them were modeled with linear, elastic, isotropic, and homogeneous properties. This assumption is employed in most of the finite element analysis in the dentistry field [10,38]. The main advantage of the model used in this study is that no simplification has been made since the geometry of the jaw is a real geometry, it has taken into account the different materials of the crown, the cement and the real geometry of the crown of the tooth.

Lee et al. [10] analyzed the stress distribution along the dental implants with different connections under oblique loads. According to our results, the MT connection obtained the highest stress value compared with the other implant connections. Balik et al. [39] also obtained a better result in the internal connection than in the MT connection under an oblique load.

According to Griffin and Cheung [7], the use of short implants was associated with low success rates, but Misch [40] and Ravidà [1] stated that extra-short implants have demonstrated good predictability and a good survival rate, and, therefore, short implants could be considered as an option for the treatment of bone atrophies. Lee et al. [10] also analyzed the risk of fracture on four short implants and they concluded that fracture was only predicted in one of the models and no damage was predicted for the other components within 10^7^ cycles.

Additionally, Neldam and Pinholt [41] stated that the use of short implants is an important option for more invasive surgeries and should be taken into account when planning areas with severe atrophies. Furthermore, Monje et al. [20] stated that changes in the surface treatment and macrogeometry of implants, as well as the development of new surgical techniques and the better knowledge of biomechanics applied to short implants increased success and survival. Another factor in survival and success rates is strictly connected to oral microbiota and systemic conditions [39,42].

Authors such as Renuard and Nissand [43] and Neldam and Pinholt [41] corroborate the idea that the characteristics of bone atrophy have a great challenge for the rehabilitation with the use of conventional implants. Therefore, the use of short implants is an option when the patient has a high level of morbidity reducing, also, the cost of the treatment.

Gross [44] and Zanatta et al. [45] corroborate that the most affected area by occlusal loads is the region of the cortical bone, adjacent to the cervical of the implants regardless of whether a bicorticalization is performed. The results obtained in this study are in agreement with these results, and clearly show that the implants and connections evaluated have a very large dissipation capacity of masticatory loads and show very few points of stress concentration in the cortical areas that could have as a consequence a reabsorption in the marginal bone crest region, which in extra short implants of 5 mm would be extremely worrying.

Renouard and Nisand [43] stated that the dissipation of masticatory loads occurs in the first millimeters around the implant neck, and that long implants do not improve the distribution of masticatory loads. The results observed in this study are in agreement with those found by the authors above, since they show that the conical macrogeometry greatly favors the dissipation of the loads regardless of the type of prosthetic connection used.

Moraes et al. [46] analyzed the influence of the prosthetic crown height under axial and oblique loading, Moraes observed that as the height of the crown increases, the stress concentration at the crest of the implant increases. The comparison between the two forms of loading shows that the oblique loading generates greater traction strength in the bone tissue. Thus, the natural increase of crowns in short implants is detrimental to the peri-implant tissue. In addition, according to the results obtained in this study, we could observe that, although the simulation was performed with long crowns, both tested connections obtained excellent results, however the short implants with the Morse taper connection obtained a better dissipation of the applied load, concentrating the loads on the prosthetic connection, and better dissipating the axial and angular loads on the implant body.

The use of extra short implants is progressively increasing in clinical practice because its efficacy has been demonstrated in the medium and long term. The use of this kind of implants avoids, among other things, aggressive surgical clinics such as bone grafts. This work affirms through numerical analysis that the behavior of extra-short implants in the stress distribution support the use of these implants in the case of bone atrophies when the patient does not want or can undergo advanced surgeries of regeneration.

## 5. Conclusions

In view of the results obtained in this study the two types of prosthetic fittings present a good stress distribution. The Morse taper connections presented better behavior than the internal in both loading configurations because they presented a lower stress concentration in the implant platform region and the marginal bone crest, thus suggesting a better preservation of the adjacent bone.

## Figures and Tables

**Table 1:** Material properties.

Material	Young’s Modulus (GPa)	Poisson’s Ratio
Feldspathic porcelain [27]	69	0.3
Enamel [28]	84.1	0.33
Cortical bone [29]	13.7	0.3
Sponge bone [29]	1.37	0.3
Zinc phosphate cement [30]	13	0.35
Cobalt chromium [31]	218	0.33
Titanium commercially pure [32]	110	0.35
Titanium grade 5 (Ti6Al4V) [32]	113.8	0.342
